# Volunteer programs, empowerment, and life satisfaction in Jordan: mapping local knowledge and systems change to inform public policy and science diplomacy

**DOI:** 10.3389/fsoc.2024.1371760

**Published:** 2024-05-30

**Authors:** Catherine Panter-Brick, Lina Qtaishat, Jannik Joseph Eggerman, Honey Thomas, Praveen Kumar, Rana Dajani

**Affiliations:** ^1^Jackson School of Global Affairs, Yale University, New Haven, CT, United States; ^2^Department of Anthropology, Yale University, New Haven, CT, United States; ^3^Conflict, Resilience, and Health Program, Yale University, New Haven, CT, United States; ^4^Taghyeer Organization, Amman, Jordan; ^5^School of Social Work, Boston College, Chestnut Hill, MA, United States; ^6^Department of Biology and Biotechnology, The Hashemite University, Zarqa, Jordan

**Keywords:** empowerment, cognitive mapping, life satisfaction, policy, refugee, participatory research, volunteer, systems change

## Abstract

**Introduction:**

Volunteering in the community is thought to provide unique benefits to people who experience limited engagement in society. In the global South, volunteer programs are often framed as empowering women and benefiting the poor, without empirical evidence or systematic investigation of what this means from a local perspective. For this reason, it is critical to represent stakeholder knowledge, understand how change happens systemically, and reduce cultural bias in scientific inquiry and public policy. As such, efforts to respect diverse narratives and problem-solving approaches are key to science diplomacy – they help us understand cultural relevance, program efficacy, and for whom a program is considered transformative.

**Methods and results:**

This study shows how Syrian refugee and Jordanian women, living in resource-poor families, articulated (i) concepts of empowerment and life satisfaction and (ii) the benefits of engaging in community-based volunteering programs. Through engaging in a participatory methodology known as Fuzzy Cognitive Mapping, women generated visual representations of these constructs and cause-and-effect reasoning. They identified several dimensions of empowerment (e.g., cultural, financial, and psychological empowerment) and several meanings of life satisfaction (e.g. adaptation, acceptance, and contentment). They also mapped connections between variables, identifying those that might catalyze change. We were specifically interested in evaluating understandings of *We Love Reading*, a program that trains volunteers to become changemakers in their local community. In simulations, we modelled how employment, education, money, and volunteering would drive system change, with notable results on cultural empowerment.

**Discussion:**

Through visual maps and scenarios of change, the study demonstrates a participatory approach to localizing knowledge and evaluating programs. This is key to improving scientific enquiry and public policy.

## 1 Introduction

Globally, many programs designed to benefit socially-disadvantaged groups, such as refugees and resource-poor women, struggle with the following question: How do we ensure the success of a given intervention? This speaks to issues of cultural relevance, program efficacy, and implementation science across diverse regions of the world. A key problem comes with asking “for whom?” is a program successful, and “by whom?” is success measured across stakeholder groups (scientists, practitioners, local actors, and global funders). It has become vital to understand diverse knowledge systems and problem-solving approaches, because interventions can run into many challenges and adopt different implementation formats. What is especially critical is to represent stakeholder knowledge, understand how change happens systemically, and reduce cultural bias in scientific inquiry. This promotes a more grounded understanding of cultural relevance—"for whom” is a program successful or transformative—and informs decision-making processes in science and public policy. In this paper, we discuss how volunteer programs in the global South have been framed to empower women and benefit the poor, without concrete understanding of local knowledge and lived experience. To address this gap, we engaged with Syrian refugee and Jordanian women, living in resource-poor households in Amman, Jordan, to map their understandings of volunteering, empowerment, and life satisfaction. Such information is important to help transcend boundaries of knowledge between lay and scientific communities, and to take action in ways that respect diverse problem-solving approaches across the world.

### 1.1 Science diplomacy and decolonizing knowledge in humanitarian contexts

Efforts to improve mutual understanding and to engage diverse stakeholders in problem-solving approaches are at the heart of science diplomacy. As argued by Ruffini (2020, p. 358), science diplomacy aims to address major global issues and to engage scientists as non-state actors in informed decision-making for the wellbeing of humanity. For example, “through the levers of international scientific co-operation and sound scientific advice to policy-making, science diplomacy would hold the power to reduce political tensions and mitigate conflicts between countries, improve mutual understanding between peoples, advance the satisfaction of common interests and contribute to global peace” (Ruffini, 2020, p. 335). Through scientific collaboration, researchers and practitioners can thus positively effect change, working to co-construct systems of knowledge in ways that explicitly connect science, global affairs, and public policy.

Yet critical readings of scientific diplomacy point to the possibility of cultural bias if mainstream scientific narratives are shaped by “the habits of mind and modes of thinking specific to the epistemic community of scientists” Ruffini (2020, p. 7). For instance, a mainstream discourse that entrenches the gaps between clinical and lay narratives of health-seeking behaviors does not improve mutual understanding of health, nor work toward designing sustained, culturally-relevant interventions. A discourse that privileges the powerful voices of academic institutions and Western knowledge systems, without attention to knowledge democracy or public engagement, entrenches power differentials and colonial practices that silence forms of knowledge production. In this respect, Hall and Tandon (2017, p. 6) have argued that “higher institutions today are working with a very small part of the extensive and diverse knowledge systems in the world.” This is important because knowledge is a powerful tool “for taking action in social movements and elsewhere to deepen democracy and to struggle for a fairer and healthier world” (Hall and Tandon, 2017, p. 13). In raising questions about whose knowledge is prioritized, how that knowledge was gathered, and how transformative change is encouraged, Hall and Tandon called for approaches that do not reinforce “the existing colonized relations of knowledge power” (Hall and Tandon, 2017, p. 7). Specifically, they argued that participatory research and community-based work can promote knowledge decolonization, leading to inclusive institutional cultures that genuinely respect diversity in knowledge narratives and problem-solving approaches.

Efforts to represent knowledge systems are certainly important when it comes to working with refugee and host communities in the Middle East. As Bennett (2018) has argued, we live in a time when alternative forms of humanitarian action need to be “re-imagined.” The protracted form of humanitarian crises, including the global refugee crisis, has called for a “rethink” and a “modernization” of international models of humanitarian assistance, in ways that put actual “people” squarely at the center of humanitarian action (Bennett, 2018). For example, new models of humanitarian assistance are questioning the underlying assumptions of power and governance in the classical, largely hierarchical, approach to working with people in need, known as “crisis humanitarianism” (Currion, 2018; Hilhorst, 2018; Panter-Brick, 2023). A network approach, for example, encourages thinking about systems, and transforming systems, rather than responding to individual need. The word “network” refers to a “system” that can bring about transformational change through supporting a diverse set of actors who have the capacity to self-organize and interconnect: in “network humanitarianism,” the flow of resources—information, technologies, partnerships, supplies—is distributed horizontally, rather than hierarchically (Currion, 2018). Similarly, within scientific communities, building “networks of networks” helps to “promote science awareness and good science practice,” allowing for “lessons from different groups to be captured and shared,” thus fostering creativity and innovation (Dajani, 2023, p. 671). Such distributive approaches to knowledge and resource flow offer the promise of building innovation, connectivity, relationality, and social inclusion in contexts of war and forced displacement.

In humanitarian contexts, taking a systemic approach is certainly important for improving decision-making with respect to health, education, work, and learning opportunities. It is also important for understanding which kinds of volunteer programs connect with the lived experience of conflict-affected groups, since volunteer programs are often designed to serve both refugee and host communities in the wake of war and forced displacement. As such, developing methods that can map diverse knowledge systems are vital to reducing cultural bias in scientific inquiry, while modeling systemic change can improve analyses and problem-solving.

### 1.2 Volunteer programs, empowerment, and life satisfaction

The United Nations has framed volunteering as a “powerful means to engage people” in ways that support the 2030 Agenda for Sustainable Development (Millora, 2020, p. 9) and global transformation. Globally, volunteering is thought to provide unique benefits to individual volunteers in terms of their social, mental, and physical health (Nichol et al., 2023), as well as foster knowledge, skills, experience, networks, and overall wellbeing (Millora, 2020, p. 15; Panter-Brick et al., 2024). Yet there has been little systematic investigation whether beneficial impacts vary across different types of volunteer programs: some are designed (top-down) by the state or international non-government organizations, others designed (bottom-up) by grassroot organizations; some rely upon hierarchical means of volunteer recruitment, others upon word-of-mouth; some require concrete time commitments, to meet project objectives, while others are more flexible over time to suit people-centered life goals. Furthermore, some articulate benefits in terms of the social, economic, and psychological empowerment of volunteers, while others emphasize life satisfaction as pathway to human wellbeing.

In the global South, a common policy narrative is that volunteering will be largely beneficial to men and women (particularly women) who are financially poor and have limited engagement with the formal sectors of society. Prevailing assumptions are that community-based work creates “model citizens” (Maes et al., 2015, p. 465) who become empowered, find satisfaction in service to others, and enact change in their community. In Ethiopia and Nepal, however, studies have highlighted that the empowerment and life satisfaction of women through volunteer work is often assumed, rather than demonstrated (Maes, 2012, p. 62; Closser et al., 2019, p. 300). This means that official narratives that community-level service brings to volunteers psychological benefits and an increased sense of moral, social, or spiritual satisfaction are “empirically questionable” (Closser et al., 2019, p. 298). In other words, these assumptions may be reflecting a stakeholder bias on the part of policy-makers, given the absence of empirical evidence regarding tangible individual-level benefits. Initiatives are thus needed to highlight the knowledge of local stakeholders, as well as clarify cause-and-effect reasoning, connections between concepts, and strength of impacts.

This undertaking is particularly important to better understand the concepts of women’s empowerment and life satisfaction, which are key to many research and program evaluations, yet remain poorly defined. In working with women in refugee and host communities, the United Nations has defined empowerment as a “process through which women […] in disadvantaged positions increase their access to knowledge, resources, and decision-making power, and raise their awareness of participation in their communities, in order to reach a level of control over their own environment” (cited in Jabbar and Zaza, 2016, p. 312). Life satisfaction, meanwhile, is associated with positive mental health, self-efficacy, and domains of wellbeing and human flourishing in refugee groups (Mohammed and Warner, 2023). Life satisfaction is used to index happiness and wellbeing, such as in the 2023 World Happiness Report, based on Gallup World Poll data (Helliwell et al., 2023). It is also measured in relation to employment or entrepreneurial activity (Lambert et al., 2020) and the degree to which individuals view themselves as achieving goals (Kahneman and Deaton, 2010).

These two concepts are rarely assessed through local analytical frameworks (Panter-Brick et al., 2024). In the development sector, for example, women’s empowerment has often been framed “as a relatively straightforward and uncontested objective” focused on agency and decision-making (O’Hara and Clement, 2018, p. 112). This candid way of framing empowerment has limited our understanding of what goals are locally meaningful and/or how they are pursued across different domains of life (Hanmer and Klugman, 2016). More often than not, the notions of empowerment, control, or agency as power, are contested. This is especially the case where women in disadvantaged positions come to interact with family norms, local institutions, humanitarian initiatives, or state power. This calls for participatory research approaches, ones that can map both explicit and tacit knowledge, built from lived experience, in local communities. Participatory research values the practice of “doing research *with* those who are typically the subjects of the research, rather than *on* them” (Vaughn and Jacquez, 2020, p. 2). They also address the needs of “under-privileged communities and groups of people who participate in research in the hope that this may bring about positive change and action” (Schubotz, 2019, p. 2).

### 1.3 Volunteer programs for women in Jordan

Jordan provides an interesting context for our study. It is the second-largest host of Syrian refugees *per capita*, after Lebanon, with about 1 in 10 of its population a Syrian refugee (UNHCR, 2023). Some 660,607 Syrians are registered as refugees or asylum-seekers in Jordan, 80% of whom live outside of refugee camps, subsisting below the poverty line. Jordan has been praised as a “model country” for espousing the Global Compact on Refugees, mobilizing investments with a view to enacting “durable solutions” for refugee and host communities (Panter-Brick, 2021). Yet political, economic, and social tensions have mired the implementation of humanitarian and development projects, including cash-based transfers, vocational training, education and healthcare services. Given the unprecedented refugee crisis the world is facing, this situation mirrors many of the challenges that governments and inter-governmental institutions are confronting globally.

Jordan is also a particularly appropriate place to evaluate interventions based on empowerment and life satisfaction. In Jordan, the government has made women’s social and economic empowerment a national priority, promoting women’s employment, vocational training, and entrepreneurial capacity (Ait Ali Slimane et al., 2020; El Kharouf et al., 2021). Recent legislation has provided good opportunities for women in Jordan: “several acting organizations implement programs to empower women, providing technical assistance to the government on legislation, policies, and strategies for women’s improved access to income security and decent work” (UN Women, 2018, quoted in Haider et al., 2021, p. 2). International donors have funded Cash-for-Work public work programs since 2016, thought to be changing stereotypes regarding women’s work participation outside the home (Zintl and Loewe, 2022). State or NGO-funded vocational and volunteer programs are often specifically conceived to help women and girls step outside of traditional family structures to engage with a “peer-to-peer support mechanism and empowerment process” (Jabbar and Zaza, 2016, p. 307). Yet these programs often fail to engage a truly local understanding of empowerment. For example, because of the conservative nature of many Syrian and Jordanian communities, kinship groups enforce the expectation that women work within the home, rather than work in the local community, let alone engage in the labor force (Miles, 2002; Culcasi, 2019). In Amman, Miles (2002, p. 425) reported that “cultural and family-level constraints to women’s employment seemed more intense in (…) low-income, densely-populated areas.” Her focus group interviews in low-income settlements suggested that this was because men felt threatened by the “power” women might gain from working outside the home, such that it was more acceptable for women to take on unpaid volunteer service in the community than renumerated work.

Our study builds upon previous scientific collaborations initiated in Jordan. In 2015, we led a program evaluation in Jordan that rested explicitly upon cross-border, inter-disciplinary, and multi-sectoral work. We engaged with Syrian refugee and Jordanian youth to assess the biological, psychosocial and cognitive signatures of war and forced displacement, and to measure the impacts of a Mercy Corps-led program to boost mental health, resilience, and social cohesion (Panter-Brick et al., 2020; Panter-Brick, 2022). Results were useful for science diplomacy, in terms of supporting the work of humanitarian actors to invest in youth-focused interventions in the Middle East region, in the wake of the Syrian civil war. As a case study, the work was also useful for developing a knowledge base on how to lead robust and culturally-grounded scientific projects in humanitarian contexts; specifically, this was of interest to program officers from the National Institutes of Health (Panter-Brick et al., 2020; Mistry et al., 2021; Panter-Brick, 2022) and to program officers from Elrha, a global charity leading research and innovation in humanitarian crises (Elrha, 2023a,b). Importantly, from the perspective of the Syrian and Jordanian research team, who did the ground-level research with Syrian and Jordanian respondents, our research showed “how local and international scientists can work together not only to do better science, but to ensure that research participants are part of the process, respecting their dignity” (Dajani et al., 2021).

In 2021, we initiated an investigation of the social networks of Syrian and Jordanian women and examined how volunteering work diversified their networks outside the home, promoting feelings of psychological empowerment and social inclusion (Eggerman et al., 2023). In 2022, we used qualitative work, panel data, and a randomized controlled trial to evaluate whether volunteering activity impacted outcomes such as psychological wellbeing, empowerment, and life satisfaction for Syrian refugee women (Panter-Brick et al., 2024). In 2023, we initiated the present study. As a multidisciplinary team of scientists, we wanted to deepen an understanding of local narratives, systems thinking, and problem-solving. We were specifically interested in how women’s engagement in volunteering programs can drive systemic change in their experience. We focused attention on *We Love Reading*, a program that trains volunteers to read aloud to children in their neighborhoods and encourages women to become changemakers in their communities (Dajani, 2019; Mahasneh et al., 2021).

### 1.4 Study goals

This study had two main goals: to show how Syrian refugee and Jordanian women, living in resource-poor families, articulated (i) concepts of empowerment and life satisfaction and (ii) the benefits of engaging in community-based volunteering programs. As such, the study aimed to map local knowledge and visualize systems change from the perspective of local stakeholders. We were specifically interested in *We Love Reading* (WLR), a program that trains volunteers to read aloud to children in their neighborhoods. First implemented in Jordanian communities in Amman in 2006, and inside Syrian refugee camps in 2014, *We Love Reading* is currently active in 65 countries of the world, and has received global recognition (Dajani, 2017, p. 4; We Love Reading, 2022). It is a rare example of South-to-South and South-to-North program diffusion, aiming to support volunteers’ agency, empowerment, competencies, and wellbeing, and to create social change through socially inclusive volunteering practices. As documented in an award-winning documentary (The Neighborhood Storyteller, 2022), *We Love Reading* volunteers are instructed on how to organize read-aloud sessions for children locally, how to think creatively about time management and defend their perspectives (Hanemann, 2018). This proves important for women in financially poor and/or culturally-conservative households, who are “empowered to become changemakers” in their community (We Love Reading, 2022, p. 4).

In employing systems thinking and participatory methodologies, we hoped to demonstrate ways to decolonize scientific approaches to research and program evaluations. Such evaluations often deploy measures in global surveys without adequate understanding of local knowledge or adequate translations of underlying constructs. Taking concepts from one culture and applying them to another culture, without careful assessment of face validity or cultural relevance, is a colonial approach to scientific research that needs attention. Assuming that concepts for one group of stakeholders, such as scientists and policy-makers in the development or humanitarian sector, are exactly the same as those of another group of stakeholders, such as refugees or the urban poor, is also problematic. Gaps of understanding between stakeholders, when used to inform humanitarian or development programs, can lead to ineffective interventions. For all these reasons, efforts to localize knowledge—showing how people reason and what they perceive drives change in the world around them—are important for research and evaluation.

## 2 Methods

We used a method known as Fuzzy Cognitive Mapping (FCM) to capture local understandings of empowerment and life satisfaction and to map the impacts of a community-based volunteering program. We exemplified an approach which can be used by scientists and policymakers to understand knowledge systems and model change in ways that ring true to local stakeholders. Specifically, we engaged Syrian refugee and Jordanian women, living in poverty in the same urban neighborhood, in visual knowledge representation and discussions of community-level change for enhancing wellbeing, agency, empowerment and learning opportunities. Social sciences methods “have often measured degrees of “systems thinking” by exploring the qualitative and quantitative attributes of individual mental models,” elicited through the means of cognitive maps (Lalani et al., 2023, p. 02). Here, systems thinking (ST) is defined as “a mental construct that recognizes patterns and connections in a particular complex system to make the “best decision” possible given a particular goal” (Lalani et al., 2023, p. 02).

### 2.1 Fuzzy cognitive mapping

Fuzzy Cognitive Mapping (FCM) is a participatory methodology used to identify and visually represent how a group of respondents perceives a system to function. The methodology produces fuzzy cognitive maps, which graphically represent a network of factors and the causal relationships between those factors. The factors can be tangible and measurable—like education or money—or abstract and unquantifiable, such as social constructs or political ideas. FCM is an approach that combines cognitive mapping with the measurement of fuzzy logic: a cognitive map helps to visualize relationships of a binary nature (on/off connections, causal or not), while FCM captures the complexity and ambiguity inherent to many relationships. Specifically, the latter approach engages respondents to assign relative weights to causal relationships, ranging from −1 to 1 in value (Barbrook-Johnson and Penn, 2022, p. 92). This semi-quantitative characteristic of FCM enables researchers to develop scenarios comparing how different components of the system may drive systemic change. FCM provides “soft models” (Sarmiento et al., 2020) of local knowledge, as well as “simplified mathematical models” (Gray et al., 2012) to indicate causality and understand system dynamics.

FCM is thus a tool for understanding systems from the vantage point of different stakeholders (Gray et al., 2015). The methodology has been used over the last two decades across disciplines, including social sciences, medicine, business, and natural sciences (Papageorgiou and Salmeron, 2013; Barbrook-Johnson and Penn, 2022). For example, researchers have employed FCM to examine the system of environmental factors that impact human wellbeing and health (Wildenberg, 2009), as well as to map how different stakeholders might respond to the impacts of climate change (Reckien et al., 2011). FCM can be used for different purposes: as a method to collect, model, and make inferences from respondent-led data; or as a tool for learning and to facilitate group participation and discussion (Barbrook-Johnson and Penn, 2022, p. 92). In FCM, participants play an active role in generating maps that represent their knowledge and perspectives, by taking part in a focus group-style discussion and contributing to the development of the map in real-time. As argued by Sarmiento et al. (2020, p. 125), the maps “offer a visual language to present and discuss indigenous knowledge and to incorporate participant voices in research and decision-making.” The maps describe different knowledge systems in ways that offer “a transparent and systematic way to organize and to summarize” information despite language, education, or intercultural differences. In health research, they are a “tool for marginalized communities to communicate their way of seeing things to health authorities and to open discussion about health initiatives” (Sarmiento et al., 2020, p. 138). That reasoning may apply to diverse research areas, including the social sciences, as participants may use the maps to understand their own perspectives on the issue at hand, communicate those perspectives, and identify entry-points for systemic change.

### 2.2 Fuzzy cognitive mapping sessions in Jordan

Our target group of participants were financially poor women, both Syrian refugees and host-community Jordanians, living in East Amman, Jordan. We partnered with *Taghyeer*, an organization registered with the Ministry of Social Development and the Ministry of Culture in Jordan, and with five community-based organizations (CBOs) who reached out by phone to women registered in their database, inviting them to participate in the study. Eligible women were 18 years and above, Syrian or Jordanian. Meetings were then scheduled in each CBO, during which written informed consent was secured from respondents. Our study received formal approval from the Prime Minister’s Office of Jordan and ethics approval from the Institutional Review Board of Yale University.

We conducted four FCM sessions (21st May to 11th August 2023) with a total 37 women (*n* = 20 Syrians and 17 Jordanians). Two of these sessions were with Jordanians, two with Syrians. Each session, of 8–10 participants, lasted between 2 and 3 h and was tape-recorded. Each session was led by a bilingual facilitator (LQ), who was assisted by one note-taker; both were Jordanian women with extensive experience in mixed-methods health research. Respondents were diverse in age (21–57 years) and education (having no formal schooling to university). In the last 6 months, just half (56.75%) had been volunteering in the community on a weekly, monthly, or yearly basis: reading to children in the neighborhood; feeding the poor; helping orphans or sick people; cleaning neighborhood streets, mosques, or parks; or helping local organizations as a counselor, instructor, or social worker. Only two women had (part-time) paid jobs. All respondents came from poor households earning between 50 and 400 Jordanian Dinars per month (the threshold used to denote the poorest tenth of Jordan’s population is 350 Jordanian Dinars ($500 USD) per month; UNICEF Jordan, 2019; Hastings et al., 2021).

In each session, we followed the approach outlined in Table 1 (as discussed by Özesmi and Özesmi, 2004; Papageorgiou and Salmeron, 2013; Gray et al., 2014; Gray et al., 2015). To guide each session, the facilitator asked: What do empowerment and life satisfaction mean for you? What factors negatively or positively interact with empowerment and life satisfaction? How are these factors connected with each other? Since we were specifically interested in discussing *We Love Reading* as a community-level intervention, we followed-up with questions such as: What is the impact of *We Love Reading* on the system and how does WLR impact dimensions of empowerment and life satisfaction?

**Table 1:** The approach of fuzzy cognitive mapping.

Step 1: Introduction	• The facilitator establishes the discussion with the participants, introducing the stakeholders and outlining the research questions • In our study, the facilitator introduced the aim of the fuzzy cognitive mapping methodology: to identify local meanings of empowerment and life satisfaction, and map factors of influence. Specifically, we asked the group, in Arabic, what does the sense of empowerment and life satisfaction mean for you?
Step 2: Mapping factors	• The facilitator asks the participants to identify the factors that will be included in the mental model. The factors include the initial concepts under study and, subsequently, the factors that interact with them, represented as $A_{1},A_{2}\subset A_{n}$ . Through an open-ended discussion, the participants arrive at $A_{n}$ , which the facilitator records • In our study, the facilitator asked participants to identify the meanings and types of empowerment and life satisfaction. The facilitator then opened discussion of factors of influence, to characterize the complete mental model of the community related to empowerment and life satisfaction Specifically, we asked the group, in Arabic, what are the factors that can increase, or decrease, your levels of empowerment and life satisfaction?
Step 3: Mapping influences	• The facilitator asks the participants to identify the causal connections between the factors $A_{n}$ . The map begins to take shape. In this step, the participants may identify new factors they had previously missed • In our study, the participants identified positive and negative causal connections
Step 4: Assigning strength	• The facilitator asks the participants to establish the relative strength of each causal connection. The strength of influence can be articulated in several ways, including qualitatively (such as “weakly negative” and “very strongly positive”) or quantitatively (from −1 to 1) • In our study, the facilitator asked participants to assign an influence strength ranging from very negative (−1) to very positive (+1)
Step 5: Simulation and analysis	• Following the session, the analyst uses software to create simulations and analyze the mental maps. In the first stage, the analyst may study the factors, direction and strength of influences, and relative significance of the factors. The connectivity measures show how the map is interconnected. For example, the analyst counts the strength of both incoming and outgoing connections to a variable, which indicates its relative importance or centrality. The analyst also identifies the driver variables, which are those that are influencing the system (positive outgoing connection) without being influenced (zero incoming connections) by other variables in the system. In the second stage, the analyst may run simulations and study the impact on the system • In our study, we used Mental Modeler, a free, online interface found at www.mentalmodeler.org (Gray et al., 2013). For each map generated by participants, we analyzed the causal connections between empowerment, life satisfaction and factors of influence. We then ran simulations on the mental maps, in isolation and in aggregated form, to simulate the relative impact of volunteering, paid work, education, and money on the system. We also analyzed the transcripts of participants’ discussion to understand concepts and causal reasoning

**Figure 1:**
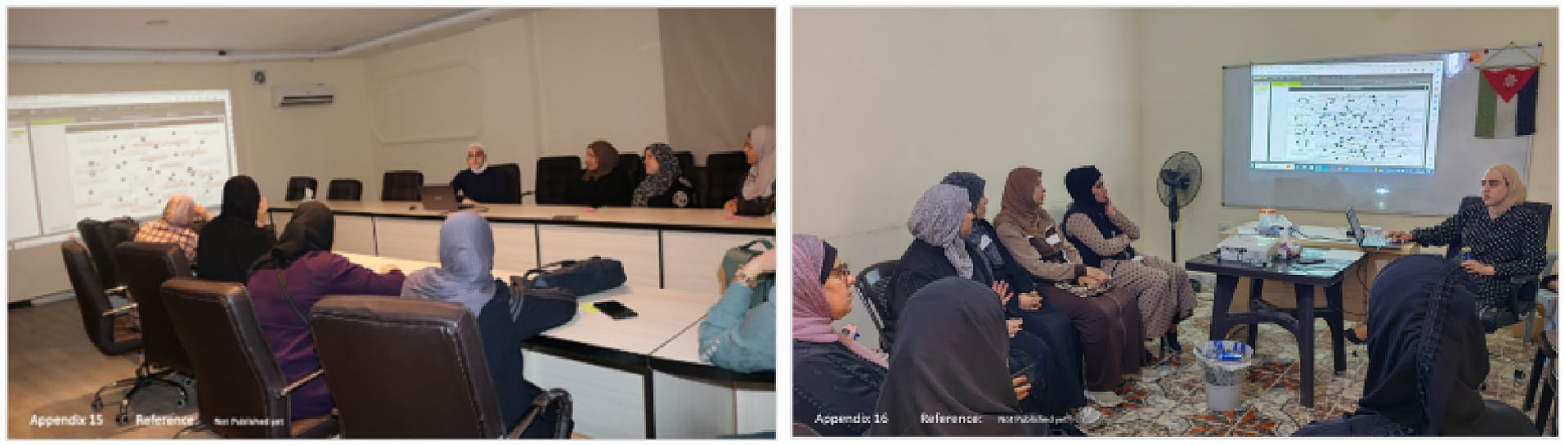
Example of two sessions during which women discussed local concepts and established connections; women placed themselves in a semi-circle, around a table or around the room, while the facilitator inputted the variables, connections, and the direction and relative strengths of connection, online. Photos reproduced with permission (copyright, Taghyeer Organization).

To facilitate the discussion, women sat in a semi-circle, in a room equipped with a laptop and projector (used by the facilitator), such that everyone in the group could see a projection of the laptop screen on the wall, as illustrated in Figure 1. Using Mental Modeler,^1^ the facilitator inputted the factors, directional influences, and values into the online tool during the course of the session. In doing so, the participants could see the fuzzy cognitive map being created in real-time. The entire discussion, in Arabic language, was recorded so that knowledge generated over the course of the session could be used in the final analysis. For example, participants explained what different types of empowerment and life satisfaction meant for them, in open discussion, and how engaging in *We Love Reading* compared to engaging with other volunteering initiatives, or having access to other resources or forms of support.

### 2.3 Analyses

We examined the women’s narratives, using inductive thematic analysis (Braun and Clarke, 2006) to group together concepts of similar meaning. We carefully recorded the examples proffered in group discussion to explain why each specific concept constituted a different idea, clarifying its relevance to different aspects of their life experience. Two co-authors (LQ, RD) worked with the Arabic-language recordings, and another two co-authors (PB, HT) with English translations. We convened as a team, using video conferencing to tabulate the different dimensions (meanings and types) of theoretical constructs and to review examples, namely the specific instances of how women discussed volunteering, empowerment, and life satisfaction.

Next, we formally compared the maps from each sessions, identifying which connections had been mentioned in a single or multiple maps, and pinpointing the relative (negative or positive) strengths of influence. We used Mental Modeler and another free online tool, Gephi,^2^ to help with visual analysis. For example, we digitally highlighted the causal relationships of interest, examining all unidirectional arrows that represented the causal influence of volunteering activities on empowerment and life satisfaction. One co-author (LQ) then used the software to create scenarios, or simulations, to analyze how the system responded to maximizing or minimizing the relative value of factors of interest. The research team used video conferencing to facilitate an extensive discussion all scenarios, generated for each of the four sessions in turn.

## 3 Results

### 3.1 Deconstructing empowerment and life satisfaction

We asked women to identify what empowerment (التمكين) and life satisfaction (الرضا عن الحياة) meant for them; specifically, the participants identified different meanings and types of these two concepts. Tables 2, 3 summarizes this information across our four groups of respondents.

**Table 2:** Deconstructing the meanings and types of empowerment, as explained by women in four sessions of fuzzy cognitive mapping discussion.

	SESSION 1	SESSION 2	SESSION 3	SESSION 4
**Empowerment meaning**				
Ability	✓	✓	✓	✓
Achievement		✓		
Acquiring a right		✓		
Awareness			✓	
Capability			✓	
Freedom		✓	✓	
Having a role in				✓
Keep firm	✓			
Opportunities		✓	✓	✓
Proof of existence		✓		✓
Reaching goals		✓		
Respectful treatment	✓			
Self-confidence	✓	✓	✓	✓
Self-reliance	✓	✓		
Social cohesion	✓			
Strength	✓	✓		
Strong personality				✓
Willingness and determination	✓	✓		✓
**Empowerment type**				
Actions empowerment				✓
Cultural empowerment		✓	✓	
Family empowerment	✓			
Financial empowerment	✓	✓	✓	✓
intellectual empowerment				✓
Managerial empowerment	✓			
Morale-boosting empowerment		✓		
Psychological empowerment				✓
Self-empowerment	✓	✓		
Social empowerment		✓	✓	

**Table 3:** Deconstructing the meanings and types of life satisfaction, as explained by women in four sessions of fuzzy cognitive mapping discussion.

	**SESSION 1**	**SESSION 2**	**SESSION 3**	**SESSION 4**
**Life satisfaction meaning**				
Acceptance	✓	✓	✓	
Adaptation	✓	✓	✓	✓
Belief in destiny				✓
Contentment	✓	✓	✓	✓
Fulfillment		✓		
Happiness	✓			
Patience and resilience				✓
Peace of mind				✓
Praising Allah			✓	
Trust in Allah		✓		
**Life satisfaction type**				
Psychological satisfaction	✓			
Satisfaction with oneself		✓		
Satisfaction with Allah		✓		
Social satisfaction		✓		
Satisfaction with physical health				✓
Satisfaction with family				✓
Satisfaction with community				✓

In each session, women identified at least 6 meanings of empowerment, ranging from having “opportunities” to take action in their lives to showing “willingness and determination.” In all four sessions, they explained that notions of “ability” and “self-confidence” were fundamental meanings of empowerment. They gave specific examples, from lived experience, showing how these components were distinct, yet interrelated, aspects of empowerment. Notions of ability and achievement rested on the freedom “to be able to do a certain thing, […] address a specific subject [or] act in a certain situation,” as well as “achieve something on your own will.” Women thus expressed empowerment as reaching their goals, being self-confident, self-reliant, and strong with or without the support of other people; in essence, empowerment meant having the self-confidence, ability, and strength to act independently.

Women also identified 10 types of empowerment—notably financial empowerment, but also cultural, self (personal), and social empowerment. Financial empowerment had to do with possessing (economic) “resources” that a woman could manage the way she wanted and led her to “running a project, … [and] being employed.” Women spoke of cultural empowerment in terms of having opportunities “to learn, to study, to educate oneself, … to become productive in the community.” Self-empowerment was to be (intrinsically) self-confident and determined regarding decision-making, else to (extrinsically) result from a boost of morale which led to a stronger resolve to take on a project of one’s own. Social empowerment was associated with the “people around you.” Specifically, women stated that “social empowerment can get something out of you that you did not know is there,” achievable through “the people around you… family… and friends.”

During the sessions, women discussed 4 to 5 different meanings pertaining to life satisfaction. All respondents saw that being “life-satisfied” meant feeling “contentment” about what one has in life, as well as showing “adaptation” in coping with different challenging situations. On a psychological level, they noted the importance of “peace of mind” as well as “patience and resilience,” leading to feelings of happiness and fulfillment, and the directive of “not to burden yourself with what is beyond your ability.” On a spiritual level, these women highlighted the need of “acceptance” (of life’s reality) and a “belief in destiny.” The notions of “contentment” and “acceptance” in life satisfaction stemmed from faith that led one to be “content with what Allah gave a person, like money, children, and a husband,” as well as acceptance “of what is meant for a person, and what Allah wrote for a person,” requiring a state of gratification and “praising Allah.”

Women also differentiated satisfaction with oneself, with Allah (the Creator), with one’s family, community, physical health, and social relationships. In terms of community-level satisfaction, women explained the importance of “the surrounding community, working environment, and the people you are dealing with.” In terms of social satisfaction, they explained being “satisfied with relationships with other people.” Women mentioned health explicitly, in terms of psychological satisfaction and satisfaction with physical wellbeing, expressing that “health is one of the blessings that makes you satisfied with your life.”

We also asked women what factors interacted with such dimensions of empowerment and life satisfaction. The factors they identified are shown in Table 4. Having a controlling husband, family support, money, and work were mentioned most frequently. During sessions, women proffered either volunteering (in general) or *We Love Reading* (in particular) as an important factor of influence; in one session where they did not mention *We Love Reading* by name, the facilitator asked about this program specifically, because this was the intervention of interest.

**Table 4:** List of factors that women identified as driving empowerment and life satisfaction, as explained by women in four sessions of fuzzy cognitive mapping discussion.

**Factors**	**SESSION 1**	**SESSION 2**	**SESSION 3**	**SESSION 4**
Affinity and love				✓
Children			✓	✓
Controlling husband (bad, imperfect)	✓		✓	✓
Discrimination				✓
Domestic violence				✓
Education	✓			✓
Emptiness of life	✓			
Failure	✓			
Family support	✓	✓	✓	
Friends	✓			
Hard life circumstances	✓			
Ignorance			✓	
Illness	✓		✓	
Loss			✓	
Mental and physical health		✓		
Mental health	✓			
Money	✓	✓	✓	✓
Morals and ethics		✓		
Negative traditions and customs			✓	✓
Nepotism (Wasta)				✓
Poverty			✓	
Recreation	✓			
Refugee status			✓	
Safety and security				✓
Unemployment			✓	✓
Volunteering (in general)			✓	✓
Volunteering (*We Love Reading* specifically)	✓	✓	✓	
Work	✓	✓		✓

### 3.2 Structural analysis of the cognitive maps

**Figure 2:**
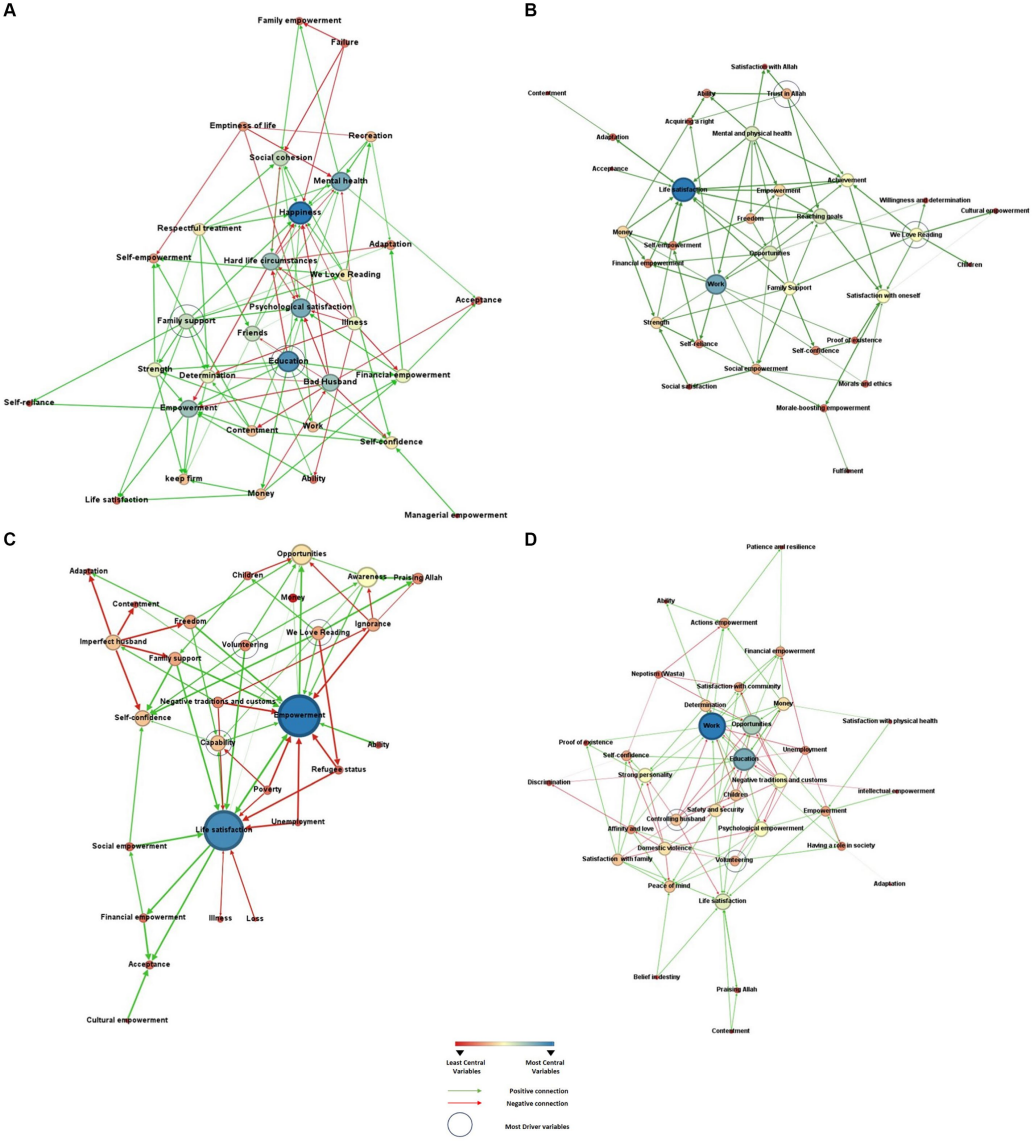
Four cognitive mental maps generated by women during the mapping sessions, based on the centrality of the variables, and identifying the variables driving change in the system. **(A)** Map 1. **(B)** Map 2. **(C)** Map 3. **(D)** Map 4.

The maps that participants generated are shown in Figures 2A–D. These maps order the variables according to their centrality—namely, the total absolute value of all incoming and outgoing connections, understood as indicative of the relative importance of that variable to the system, in the minds of the participants. The size and color of the circles in the maps denote the centrality of each variable. In addition, the maps show the driver variables—variables that only influence the system, and are not themselves directly influenced. The most important driver variables are denoted by a double circle. Finally, the positive and negative causal connections between variables are shown as green and red directional arrows.

In Figure 2A, we see that education and happiness are the most central variables (denoted in blue or green), alongside mental health and psychological satisfaction. Family support—a driver variable—positively impacts the dimensions of empowerment (namely, self-empowerment, strength, determination, self-reliance) and dimensions of life satisfaction (adaptation, contentment, happiness, and life satisfaction as a generic construct). Education—also a driver—positively impacts strength, determination, financial empowerment, self-confidence, and the generic concept of empowerment; it also directly impacts psychological satisfaction. The map shows that women saw that having a “bad husband” negatively impacted their sense of empowerment, their feelings of happiness, their levels of contentment and acceptance, as shown by red arrows.

In Figure 2B, we see that work and life satisfaction are the most central variables. Work impacts dimensions of empowerment, such as financial empowerment, self-reliance, proof of existence, self-confidence, and social empowerment; it impacts life satisfaction only through having money (the arrows go from work to money, and from money to life satisfaction). As driver variables, “trust in Allah” and *We Love Reading* are two main variables positively driving change in the system. For example, they both positively influence levels of “achievement” and “reaching goals.” “Trust in Allah” also influences ability and “acquiring a right” (as women get their strength from Allah, they can fight for their rights). *We Love Reading* impacts cultural empowerment, willingness and determination, and satisfaction with oneself. Specifically, women felt that contributing to the community through *We Love Reading* volunteering activities made them feel more positive and satisfied with their self-image and role in life.

In Figure 2C, empowerment and life satisfaction are the most central variables: these variables are highly-impacted by the system, as seen by the many incoming arrows. This map shows three driver variables, as denoted by a double circle: negative traditions and customs, *We Love Reading*, and volunteering in general. Interestingly, we see that *We Love Reading* decreases the negative consequences of being a refugee (their “refugee status”). We see that volunteering has a positive influence on awareness, life satisfaction, opportunities, and self-confidence. Like Map 1, having an “imperfect husband” (meaning, one who is controlling) negatively impacts a women’s freedom and self-confidence (defining empowerment), and contentment and adaptation to life (defining life satisfaction).

Lastly, in Figure 2D, work, education and opportunities are central variables, while having a controlling husband and volunteering are the system’s drivers. Specifically, women said that having a “controlling” husband and experiencing domestic violence decreased satisfaction with one’s family, and weakened their personality. Women also said that volunteering activities helped decrease domestic violence and resist “negative traditions and customs.”

Table 5 shows the indices characterizing each map, as a system of directional, weighted and causal connections. The four maps had a density ranging from 0.074 to 0.099: this means that only 7–9.9% of the possible connections between all variables were actually generated or formed by the respondents. This is a low to moderate map density, which tells us that implementing systemic change (through the manipulation of drivers) will be moderately difficult. While women saw many factors affecting their lived experiences, their mental models indicate only a low to moderate possibility of manipulating or changing the system.

**Table 5:** Graph theory indices for the mental maps shown in Figures 2A–D, generated by women in Fuzzy Cognitive Mapping sessions.

Map	No. of variables	No. of connections	Density	Average connections per variable	No. of driver variables	No. of receiver variables	No. of ordinary variables	Complexity
Map 1	32	98	≈0.099	≈3	6	8	18	≈1.33
Map 2	33	78	≈0.074	≈2	5	5	23	1
Map 3	28	66	≈0.087	≈3	8	3	17	≈0.375
Map 4	35	108	≈0.091	≈3	5	5	25	1

### 3.3 Scenarios

**Figure 3:**
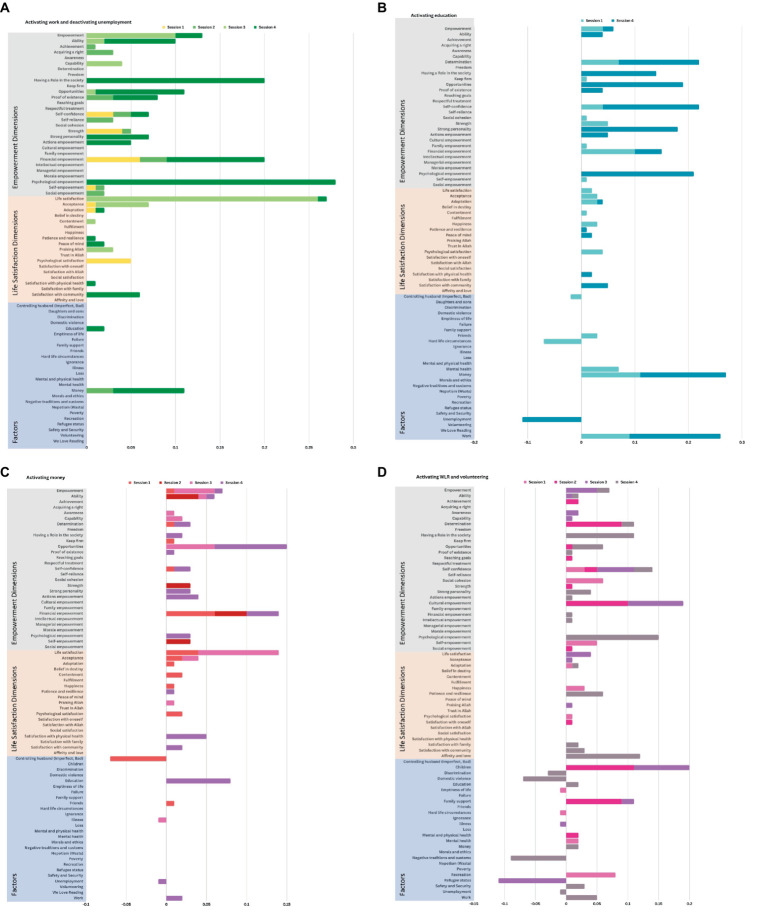
Scenarios simulating the relative impact of volunteering, paid work, education, and money on the mental map system. **(A)** Paid work: Simulated impact of increasing the value of paid work to its maximum (+1) and decreasing the value of unemployment to its minimum (−1). For example, we observe a relative positive increase in financial empowerment. **(B)** Education: Simulated impact of increasing the value of education to its maximum (+1). For example, we observe a relative positive increase in money and work. Education was only mentioned as a variable in session 1 and session 4. **(C)** Money: Simulated impact of increasing the value of money to its maximum (+1). For example, we observe a relative positive increase in opportunities, financial empowerment, and life satisfaction. **(D)** Volunteering: Simulated impact of increasing the value of volunteering (Volunteering in general and We Love Reading) to its maximum (+1). For example, we observe a relative positive increase in cultural empowerment.

We ran network simulations to understand system change. Using the Mental Modeler software, we manipulated the value of chosen variables to predict the impact the changes would have on the system in four different scenarios. We did so by changing the value of a selected variable to its maximum (+1) and then observed how this change affected other variables in the system. In Figure 3, we aggregated the information of all four sessions to generate a comparison of the relative importance of the impacts of (i) paid work, (ii) education, (iii) money, and (iv) volunteering.

In the first three scenarios, we manipulated the variables associated with work, education, and money, to understand their relative influence on the system of interconnections. We see common changes in the system: in all three scenarios, we observe a relative positive increase in financial empowerment. In the first scenario (Figure 3A: work), we see that increasing levels of paid work impacts six dimensions of empowerment (empowerment as a general concept, ability, having a role in society, opportunities, financial empowerment, and psychological empowerment) as well as the generic concept of life satisfaction. It also increases the resource of having money. In the second scenario (Figure 3B: education), we see that increasing levels of education impacts four dimensions of empowerment (determination, financial empowerment, psychological empowerment, and self-confidence), as well as opportunities and money. We also see a small impact of education on dimensions of life satisfaction. In the third scenario (Figure 3C: money), we see that money increases several dimensions of empowerment (general empowerment, ability, financial empowerment, opportunities), and also increases life satisfaction. We also see that when women have money, and therefore are relatively more financially independent, we see a decrease in the negative influence of a controlling husband.

In the last scenario (Figure 3D: volunteering), we manipulated the variables associated with volunteering in the community: increasing the values of “volunteering” in general and “*We Love Reading*” in particular, to their maximum (+1). We observed a relative positive increase in cultural empowerment and self-confidence (two dimensions of empowerment), as well as increasing interactions with children (due to the nature of the *We Love Reading* program, which has women read to children in the community). We also see a relative decrease in discrimination, domestic violence, and negative traditions and customs. We then compared the impacts of general volunteering vs. *We Love Reading* programs (scenarios not shown). For women, volunteering programs created more “opportunities,” enabled them to “have a role in the society” and enhanced their “psychological empowerment,” while *We Love Reading,* specifically, contributed to “cultural empowerment,” building positive relationships with children, and helping win “family support.” Interestingly, volunteering helped reduce domestic violence and the constraints of negative traditions and customs, while *We Love Reading* specifically helped mitigate the circumstances of being a refugee, by creating spaces for enjoyment, relaxation, and relief from life’s hard circumstances.

## 4 Discussion

Volunteer programs are designed to benefit key stakeholders in society, including those who volunteer in their local community. They may deploy top-down or bottom-up approaches to mobilizing people to serve in their local communities. A key problem comes from framing volunteer programs as empowering women and benefiting the poor without concrete understanding of how these stakeholders perceive the world around them. This speaks to why it becomes critical to map knowledge representation and model systems change, from the perspective of local stakeholders, to ensure cultural relevance and inform decision-making in science and public policy. The critical issue is to understand “for whom” different programs are successful, or indeed, transformative.

Our study demonstrates a participatory approach to modeling system change, via scenarios that can (i) visualize causal connections and (ii) inform different forms of interventions. Specifically, we described women’s culturally-embedded knowledge regarding empowerment and life satisfaction, and we analyzed cause-and-effect reasoning about how volunteering changed their life experience. In this way, the study localized knowledge and encouraged systems thinking to inform public policy and science diplomacy. We discuss both aspects of the study below, hoping to crystalize “lessons learnt” and build capacity frameworks for problem-solving with refugee and host populations.

### 4.1 Localizing knowledge and informing decision-making

Efforts to localize knowledge pertaining to women’s empowerment and life satisfaction are important, given that such concepts are poorly defined, especially in the Arab world. Engaged in fuzzy cognitive mapping, women articulated what a sense of empowerment and life satisfaction meant to them, and which factors negatively or positively affected these states of being, through the prism of their own experiences. Interestingly, women understood empowerment as encompassing cultural, financial, personal, and psychological dimensions. This is not far from the United Nations’ definition of empowerment we cited earlier, namely a “process through which women […] increase their access to knowledge, resources, and decision-making power, and raise their awareness of participation in their communities” (see Jabbar and Zaza, 2016). Our respondents, however, framed their access to decision-making power in ways that were highly *relational*, going beyond notions of individual-level agency, power, or control. Women spoke of willingness and determination to be “visible in the community,” taking actions to “show” others what they were able to do. They were empowered through developing “ability” as well as “achievement,” in ways that offered proof—to themselves and others—of having the “freedom [to] act in a certain situation” and “achieve something on your own will.” Importantly, women who were Syrian refugees or poor Jordanians had to fight to acquire such freedom in decision-making, by asserting themselves and by enlisting social support. They had to overcome the influence of “negative customs and traditions” and find ways to “boost their morale” and fight for their goals with determination. Thus, one respondent explained, “morale means self-confidence, I work, and I do not care what other people say.” Women also relied on social networks, being quick to identify that social empowerment was achieved through “the people around you… family… and friends.”

Women’s narratives were more *holistic* than many forms of discourse that globally characterize empowerment and life satisfaction. In their eyes, empowerment was not limited to financial means or psychological feelings: respondents spoke of cultural empowerment, namely, having opportunities “to learn, to study, to educate oneself, … to become productive in the community.” In their eyes also, life satisfaction was not reducible to happiness and contentment. They intertwined faith and family in narratives of life satisfaction, explicitly linking contentment to acceptance of one’s destiny and “what Allah wrote for a person,” and saw that having an “imperfect husband” weakened their personality, impacted their sense of freedom and self-confidence, and affected their levels of contentment and acceptance.

Because women mapped connections between the variables, and assigned relative weights to perceived causal connections, their visual maps represented not only local constructs but also potential drivers of change. We compared four different scenarios, using the Mental Modeler software to manipulate the value of select variables and predict relative change within the model. Our variables of interest were paid work, education, money, and volunteering activity. These are tangible factors, amenable to programmatic intervention and public policy. They are also readily shaped through interventions in a local community, and commonly thought to impact human dignity and quality of life. We saw that access to resources such as employment, money, or education was impacting respondents on a personal level, in terms of their sense of ability and self-confidence. We also saw that volunteering, generally, and *We Love Reading*, specifically, impacted women at individual, family and community levels; for example, they boosted cultural empowerment, lowered domestic violence, and counteracted negative traditions and customs. Specifically, our results suggest that, for these groups of Syrian and Jordanian women, increasing access to volunteering programs such as *We Love Reading* did create more “opportunities” for learning and social interactions outside the home, enabling them to “have a role in the society” and enhancing both “psychological empowerment” and “cultural empowerment.” *We Love Reading* specifically helped to build positive relationships with children, and win “family support.” This is not to say that women saw themselves as agents or catalysts of change: they generated maps of low to moderate density, which indicated a low to moderate possibility of implementing systemic change.

Gaining a more locally-accurate understanding of the nature and impact of volunteering programs can have important, practical applications for science diplomacy and public policy. Indeed, the many different forms of formal and informal volunteering programs in existence, globally, have called for more comparative and theoretical work (Piliavin and Siegl, 2015). This would generate evidence to guide “the development of volunteering as a public health intervention” (Jenkinson et al., 2013, p. 773). It would also guide the design of volunteering programs to best serve the learning, work, and social opportunities of people in historically marginalized communities (Panter-Brick et al., 2024). Grassroot programs such as *We Love Reading*, for example, have grown through horizontal diffusion, through informal word-of-mouth recruitment and individual initiative, rather than through formal channels of institutional scale-up. Our study gives a sense of how to design culturally-relevant, locally-grounded, community-based programs, as well as how to evaluate them in a locally-informed manner.

### 4.2 Study strengths and limitations

In terms of strengths, the study offers unique topical and methodological contributions, and was conducted with groups of women who are not usually consulted about program benefits or folded into problem-solving. With FCM, it uncovered local meanings and factors of influence relevant to system change. As Sarmiento et al. perceptively argued (Sarmiento et al., 2020, p. 137), “with these methods in hand, Western epistemological frameworks need not go unchallenged in intercultural settings.” Importantly, the women engaged in this study appreciated this methodology. They saw that the maps represented their local knowledge, and they felt positive about the sessions: they reflected that mapping sessions were a form of group therapy, because they were given the chance to talk about their experiences and life story. They were happy with the exercise because it represented “their map” and “their life story.”

We noted five limitations. First, our analyses do not describe all factors of influence, being guided by specific research interests. It has been noted that FCM methodology can be at once overly simplistic in modeling, and very complicated in representing stakeholder knowledge (Gray et al., 2012). In our sessions, we addressed both concepts of empowerment and life satisfaction at once, and did not lead with the concept of wellbeing, which we had interrogated in previous work (Panter-Brick et al., 2024). Addressing each concept separately, or wellbeing on its own, would have required more sessions during the time available for fieldwork. This is to say that additional sessions, simulation, or analysis could unpack different dimensions of the system represented in women’s maps.

Second, typical to community-based participatory mental modeling approaches, there could be issues of bias, regarding information recall or participant sampling affecting the mental models developed. For example, there may exist a bandwagon effect when some participants are more vocal than others in speaking up during the participatory model building process, with more reticent participants tending to support their vocal counterparts (Hovmand, 2014). We mitigated this with careful group facilitation, by scheduling multiple sessions, and by inviting a small number of participants to sit in comfortable semi-circles.

Third, FCM is a semi-quantitative mental modeling approach to explore complex social systems. The relative change in the values of factors during simulation may not have a rich intrinsic value, and the simulation findings are difficult to interpret in absolute terms. The most important aspect of the system to analyze is the pattern of behavior (the increase or decrease of factors during simulation, and relative change), rather than the absolute values themselves. For this reason, it is useful to be able to aggregate four scenarios into one, as we were able to do in Figure 3.

Fourth, once the variables have been identified and mapped, the FCM approach does not account for exogenous influences on the system, nor the possibility that the influence of a given factor (such as family support, or money) varies over time. This limits the insights gained from generating scenarios.

Lastly, we did not seek to map how knowledge systems differed between Syrian and Jordanian respondents. While globally, urban refugees are increasingly becoming “an indistinguishable part” of the urban poor (Hilhorst, 2018, p. 40), future work could map out more explicitly whether financially poor women, who live side-by-side within the refugee and host community, have different stakeholder knowledge. It is worth noting that studies that have mapped causal reasoning with diverse groups of stakeholders – such as professionals, urban planners, and students in studies of climate change (Wildenberg, 2009; Reckien et al., 2011)—are better positioned to identify how social actors see potential system change.

## 5 Conclusion

This study localized knowledge through a close reading of the lived experiences of a hard-to-reach group of women: Syrian refugees and Jordanian citizens living in poverty, with limited opportunities to engage in formal sectors of society. We implemented a Fuzzy Cognitive Mapping methodology, one that proved well-suited to avoiding forms of cultural and scientific bias, to decolonizing approaches to scientific advice, and to supporting problem-solving in cross-cultural contexts. Unpacking local mental maps of lived experiences, and running simulations to identify likely levers of change, can be a very useful process. It can answer critical questions such as “for whom?” different programs are successful, or indeed, transformative. It can also be a very welcome step for participants themselves, amplifying their voice and encouraging local ownership. Efforts to describe culturally-embedded knowledge and encourage a systems view of change is science diplomacy: it is key to connect scientific enquiry with public policy, through understanding the distributive flow of knowledge and resources, and improving the design of interventions.

## Data availability statement

The raw data supporting the conclusions of this article will be made available by the authors, without undue reservation.

## Ethics statement

The studies involving humans were approved by Yale University IRES Review Board (#1502015359). The studies were conducted in accordance with the local legislation and institutional requirements. The participants provided their written informed consent to participate in this study. Written informed consent was obtained from the individual(s) for the publication of any potentially identifiable images or data included in this article.

## Author contributions

CP-B: Conceptualization, Data curation, Formal analysis, Funding acquisition, Investigation, Methodology, Project administration, Resources, Supervision, Validation, Visualization, Writing – original draft, Writing – review & editing. LQ: Conceptualization, Data curation, Formal analysis, Investigation, Methodology, Project administration, Resources, Software, Supervision, Validation, Visualization, Writing – review & editing. JJE: Formal analysis, Methodology, Writing – original draft, Writing – review & editing. HT: Formal analysis, Methodology, Writing – original draft, Writing – review & editing. PK: Conceptualization, Formal analysis, Methodology, Software, Visualization, Writing – original draft. RD: Conceptualization, Formal analysis, Methodology, Project administration, Resources, Supervision, Validation, Visualization, Writing – review & editing.

## Footnotes

^1^ http://www.mentalmodeler.com

^2^ http://www.gephi.org
